# Integrated transcriptomic and metabolomic data reveal the flavonoid biosynthesis metabolic pathway in *Perilla frutescens* (L.) leaves

**DOI:** 10.1038/s41598-020-73274-y

**Published:** 2020-10-01

**Authors:** Tao Jiang, Kunyuan Guo, Lingdi Liu, Wei Tian, Xiaoliang Xie, Saiqun Wen, Chunxiu Wen

**Affiliations:** 1 Institute of Cash Crops, Hebei Academy of Agricultural and Forestry Sciences, Shijiazhuang, 050051 Hebei China; 2 Institute of Chinese Herbal Medicines, Hubei Academy of Agricultural Sciences, Enshi, 445000 Hubei China

**Keywords:** Bioinformatics, Transcription, Transcriptomics, Secondary metabolism

## Abstract

*Perilla frutescens* (L.) is an important medicinal and edible plant in China with nutritional and medical uses. The extract from leaves of *Perilla frutescens* contains flavonoids and volatile oils, which are mainly used in traditional Chinese medicine. In this study, we analyzed the transcriptomic and metabolomic data of the leaves of two *Perilla frutescens* varieties: JIZI 1 and JIZI 2. A total of 9277 differentially expressed genes and 223 flavonoid metabolites were identified in these varieties. Chrysoeriol, apigenin, malvidin, cyanidin, kaempferol, and their derivatives were abundant in the leaves of *Perilla frutescens*, which were more than 70% of total flavonoid contents. A total of 77 unigenes encoding 15 enzymes were identified as candidate genes involved in flavonoid biosynthesis in the leaves of *Perilla frutescens*. High expression of the *CHS* gene enhances the accumulation of flavonoids in the leaves of *Perilla frutescens*. Our results provide valuable information on the flavonoid metabolites and candidate genes involved in the flavonoid biosynthesis pathways in the leaves of *Perilla frutescens*.

## Introduction

*Perilla frutescens* (L.), which is a self-compatible annual herb, belongs to the family Lamiaceae. This species has been widely cultivated in China, Japan, and Korea for centuries. *Perilla frutescens* is an important medicinal and edible plant in China with medical and nutritional uses^1^. Its leaves can be utilized as a transitional medicinal herb, as a vegetable, and as a spice, and its seeds can be processed into foods and nutritional edible oils^2^. The extract from leaves of *Perilla frutescens* is composed of many chemical components containing flavonoids, polysaccharides, amino acids, and trace elements, which are mainly used in traditional Chinese medicine^3^. Flavonoids serve specific functions to the plant under specific developmental or biotic/abiotic conditions and are the first line of defense against ultraviolet rays and pathogens^4^. Previous studies reported that flavonoids have many biological functions such as anti-inflammatory, anti-oxidative, anti-diabetic, and anti-hypertensive activities^5–8^.

The biosynthesis of flavonoids is regulated by a series of enzymes related to the phenylpropanoid and flavonoid pathways^9^. Among these enzymes, phenylalanine ammonia-lyase (PAL), cinnamic acid 4-hydroxylase (C4H), 4-coumarate coenzyme A ligase (4CL), and acetyl-CoA carboxylase (ACC) catalyze the biosynthesis of phenylpropanoids. Chalcone synthase (CHS), chalcone isomerase (CHI), favonol synthase (FLS), flavanone 3-hydroxylase (F3H), flavonoid 3′-hydroxylase (F3′H), flavonoid 3′5′-hydroxylase (F3′5′H), dihydroflavonol 4-reductase (DFR), anthocyanidin synthase/leucocyanidin dioxygenase (ANS*/*LDOX), and flavonoid 3-O-glucosyltransferase (UFGT) are key enzymes that control the biosynthesis of flavonoids. The above-mentioned genes involved in the biosynthesis of flavonoids have been reported in many plant species such as *Arabidopsis* and *Ginkgo biloba*^10,11^. In addition, the expression levels of genes and transcription factors also play key roles in regulating flavonoid biosynthesis^12^. For example, a high expression of *CHS* and *F3H* genes increases the accumulation of flavonoids in citrus fruit^13^.Transcription factors of R2R3-MYB, basic helix-loop-helix (bHLH), and WD40 can form an MBW complex to regulate the biosynthesis of phenylpropanoids^14^ and flavonoids^15^.

Although the flavonoid biosynthetic pathway is well described in other plants, the molecular mechanisms regulating the biosynthesis of flavonoid are still unclear in the leaves of *Perilla frutescens*. In this study, we conducted research on the flavonoid biosynthesis metabolic pathway in the leaves of *Perilla frutescens* using metabolomic and transcriptomic approaches. We aimed to explain the quantitative and qualitative differences of flavonoids and to analyze the differentially expressed genes (DEGs) involved in flavonoid biosynthesis using bioinformatics. Our results not only provide the candidate genes but also valuable information for the metabolic engineering of flavonoid biosynthesis in the leaves of *Perilla frutescens*.

## Results

### Measurement of total flavonoid and anthocyanin contents in the leaves of *Perilla frutescens*

**Figure 1:**
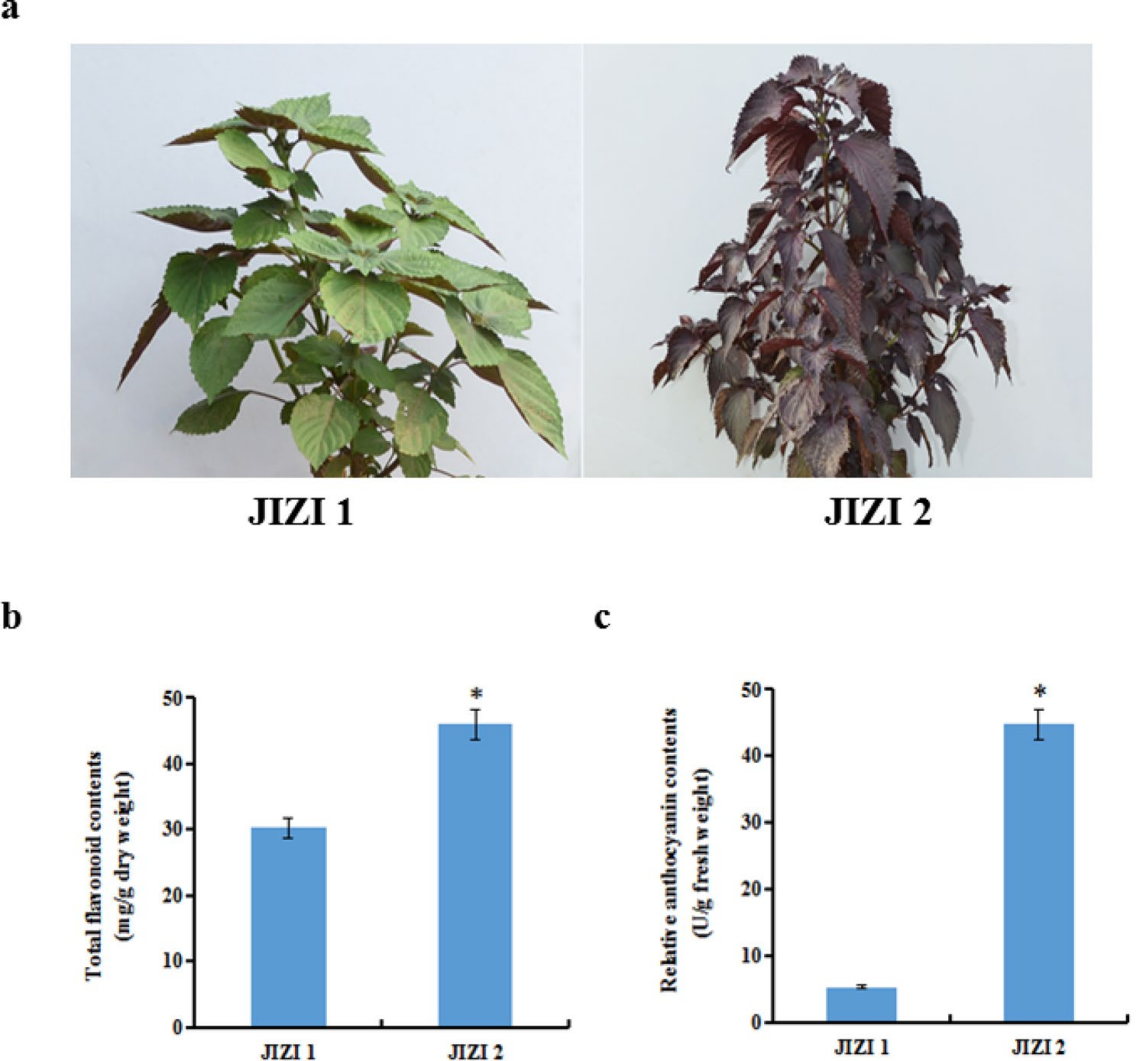
Measurement of total flavonoids and anthocyanins in the leaves of *Perilla frutescens*. (**a**) Phenotypes of two *Perilla frutescens* varieties JIZI 1 and JIZI 2. (**b**) Total flavonoid contents of JIZI 1 and JIZI 2. c Relative anthocyanin contents of JIZI 1 and JIZI 2. Each value was the mean ± standard deviation (n = 3), the star indicated the significance at the level of 0.05.

In our study, the total flavonoid and anthocyanin contents were measured in the leaves of *Perilla frutescens*. The results showed that the total flavonoid content of JIZI 2 was about 45.9 mg /g of dry weight, which was higher than the 30.2 mg/g dry weight of JIZI 1, and the relative anthocyanin content of JIZI 2 was 44.66 units/g of fresh weight, which was significantly higher than the 5.34 units/g fresh weight of JIZI 1 (Fig. 1).

### Flavonoid metabolites in the leaves of *Perilla frutescens*

**Figure 2:**
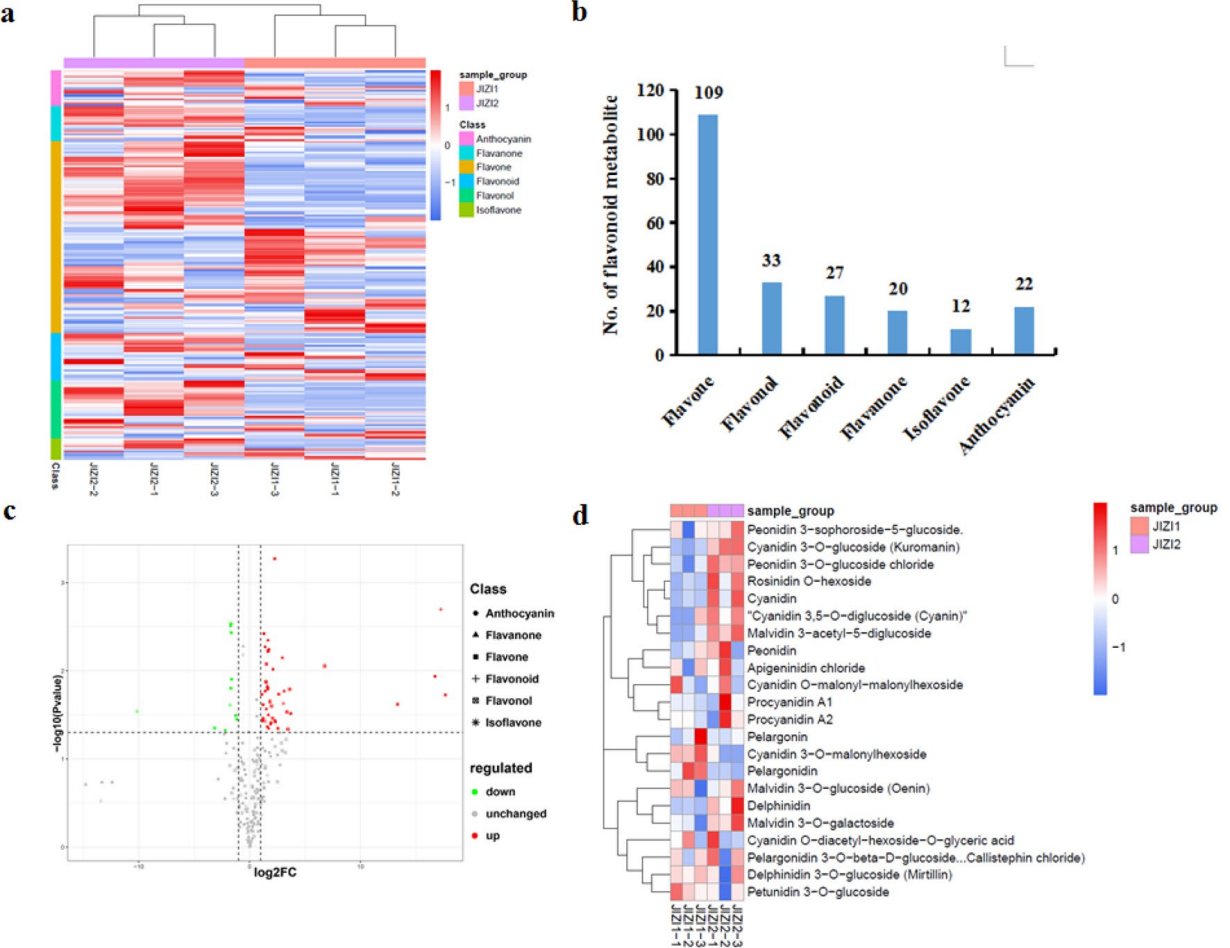
Analysis of flavonoid metabolites in the leaves of *Perilla frutescens*. (**a**) Cluster heat map of all detected flavonoid metabolites between group JIZI 1 and JIZI 2. Each sample was represented by a column, and each metabolite was represented by a row. The abundance of each metabolite was represented with different color. Red indicated high abundant metabolites, whereas low abundant metabolites were shown in blue. (**b**) Number of flavonoid metabolites in different categories. (**c**) Volcano plot of the differential flavonoid metabolites between JIZI 1and JIZI 2. Up-regulated and down-regulated metabolites were showed by red and green, respectively. (**d**) Different anthocyanin metabolites in the leaves of *Perilla frutescens*.

To compare the differential flavonoid metabolites between JIZI 1 and JIZI 2, the data obtained from UPLC/ESI-Q TRAP-MS/MS were analyzed. In this work, a total of 223 different flavonoid metabolites were identified in the leaves of *Perilla frutescens* (Table S1). The heatmap of metabolites was drawn by R software after unit variance scaling (UV), and hierarchical cluster analysis (HCA) was performed on the accumulation pattern of metabolites among different samples (Fig. 2a). The 223 flavonoid metabolites were classified into 6 categories, including 109 flavones, 33 flavonols, 27 flavonoids, 22 anthocyanins, 20 flavanones, and 12 isoflavones (Fig. 2b). Among the 223 flavonoid metabolites, chrysoeriol, apigenin, malvidin, cyanidin, kaempferol, and their derivatives were abundant in the leaves of *Perilla frutescens*, which were more than 70% of total flavonoid contents. Using the identification criterion of the absolute Log_2_FC ≥ 1 and VIP value ≥ 1, 57 (25.6%) flavonoid contents were significantly different among the 223 flavonoid metabolites, including 46 upregulated metabolites and 11 downregulated metabolites (Fig. 2c). In total, 22 anthocyanins were identified in the leaves of *Perilla frutescens*, including cyanidin, petunidin, peonidin, pelargonidin, delphinidin, and malvidin (Fig. 2d). Among them, the contents of cyanidin, malvidin 3-acetyl-5-diglucoside, and cyanidin 3, 5-O-diglucoside were high in the leaves of *Perilla frutescens*. The contents of four anthocyanins were significantly different among the 22 anthocyanins, including 3 upregulated anthocyanins and 1 downregulated anthocyanin. Among them, malvidin 3-acetyl-5-diglucoside, peonidin 3-O-glucoside chloride, and cyanidin 3-O-glucoside demonstrated significantly higher (2.16-fold to 3.02-fold) contents in JIZI 2 vs. JIZI 1, whereas cyanidin 3-O-malonylhexoside had a lower content. Malvidin, peonidin, and cyanidin could be mainly responsible for the purple leaf color of *Perilla frutescens.*

### Differentially expressed genes in the leaves of *Perilla frutescens*

**Figure 3:**
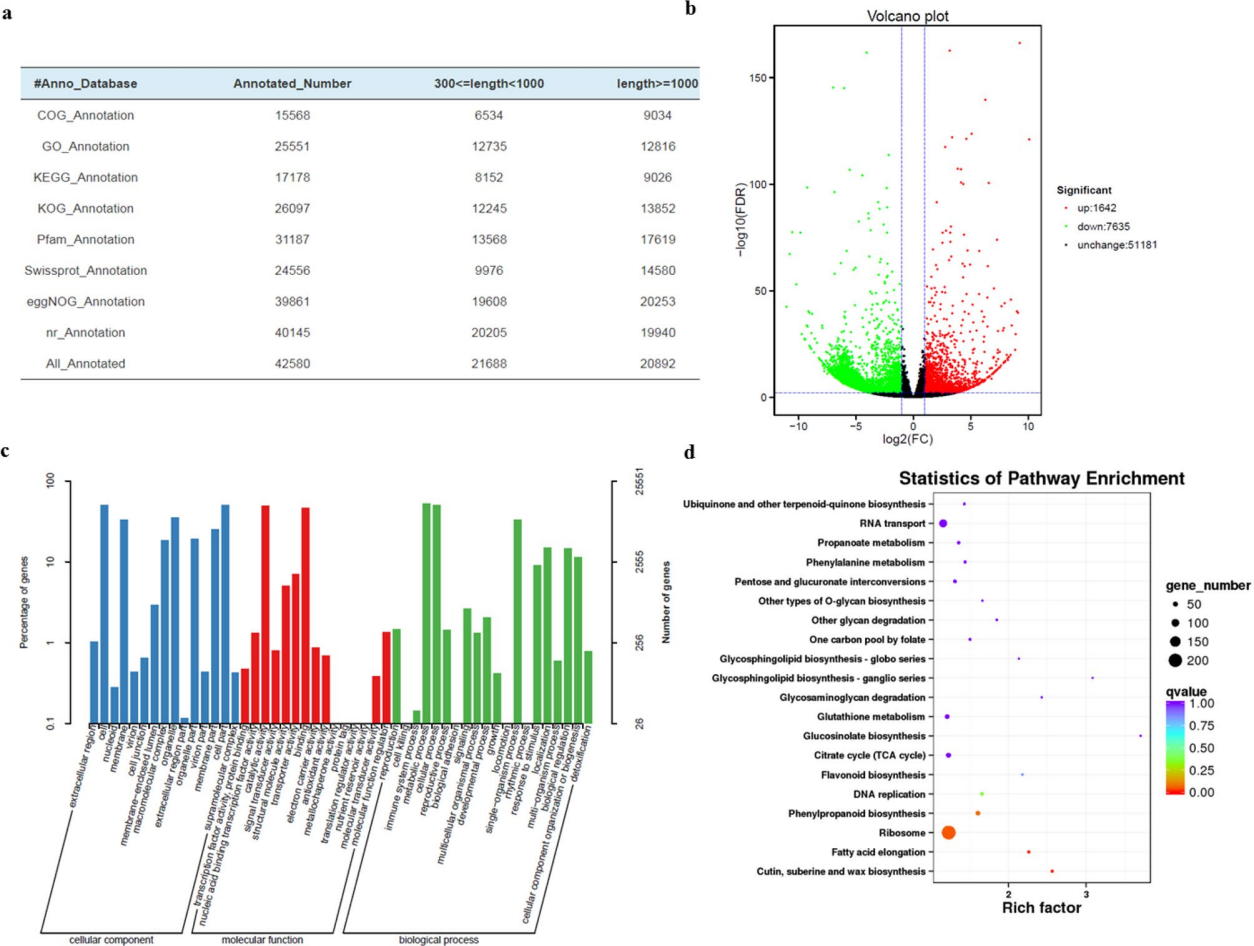
Analysis of differentially expressed genes in the leaves of *Perilla frutescens*. (**a**) Functional annotation of unigenes in the COG, GO, KEGG, KOG, Pfam, Swissprot, eggnog and Nr databases. (**b**) Volcano plot of the differentially expressed genes between JIZI 1 and JIZI 2. (**c**) GO functional classification of DEGs. d KEGG pathway enrichment of the DEGs. Significantly different genes were enriched in several metabolic processes including phenylpropanoid and flavonoid biosynthesis pathways.

To understand the molecular basis of flavonoid biosynthesis metabolic pathway, transcriptomes were analyzed to identify differentially expressed genes in the leaves. A total of 54.75 million clean reads were produced from the leaves of *Perilla frutescens*. These clean reads were further assembled into 60,458 unigenes with a mean length of 1194.19 bp using Trinity software. All unigenes were searched in the Nr, GO, COG, Swiss-Prot, egg-NOG, Pfam, KOG, and KEGG databases with the BLASTX (E-value < 1.0E-5) program for functional annotations. Among of 60,458 assembled unigenes, a total of 42,580 (70.43%) unigenes were annotated functionally based on the above-mentioned databases (Fig. 3a and Table S2). With the filter criteria of |Log_2_FC| ≥ 1 and FDR < 0.05, 9277 differentially expressed genes (DEGs) were identified in the leaves of *Perilla frutescens*, including 1642 upregulated genes and 7635 downregulated genes (Fig. 3b). In GO enrichment analysis of DEGs, 3711 DEGs out of 9277 were involved in three major GO categories, i.e., biological process, cellular component and molecular function (Fig. 3c), and 12 phenylpropanoid biosynthetic process, 29 flavonoid biosynthetic processes, 15 flavonoid glucuronidation processes, and 9 anthocyanin-containing compound biosynthetic processes were identified in the biological process category.

To understand the biological functions and gene interactions, 17,178 unigenes (including 3132 DEGs) were annotated by the KEGG database, which were divided into 126 metabolic pathways. KEGG metabolic pathway enrichment analysis using Q-value < 0.05 showed the DEGs were enriched in many metabolic processes that included phenylpropanoid biosynthesis and flavonoid biosynthesis pathways (Fig. 3d).

### Combined transcriptome and metabolome analysis revealed the biosynthesis of flavonoid in the leaves of *Perilla frutescens*

**Figure 4:**
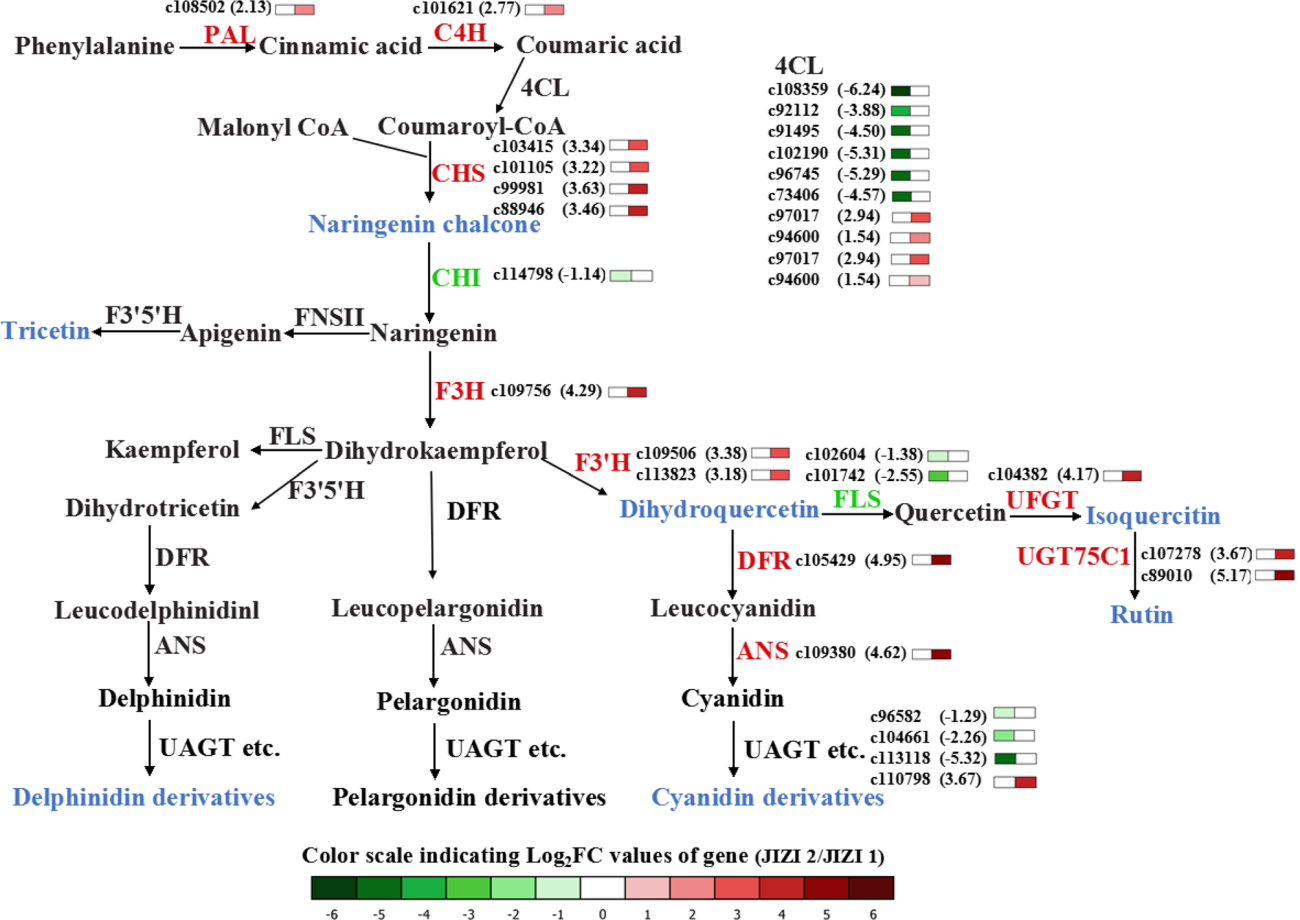
Biosynthetic pathway of flavonoids in the leaves of *Perilla frutescens*. Heat map showed the changes of transcripts and metabolites in flavonoid biosynthesis. Upregulated genes were in red, and downregulated genes were in green. Blue indicates a higher content of metabolites in JIZI 2 than in JIZI 1.

We combined the analysis of transcriptomic and metabolomic data to understand the flavonoid biosynthesis pathway in the leaves of *Perilla frutescens*. The result demonstrated that a large number of flavonoids were detected in the leaves of *Perilla frutescens*. The identified flavonoids were present in the flavonoid biosynthesis pathway (Fig. 4). From the pathway, we found that naringenin chalcone, tricetin, syringetin, dihydroquercetin, isoquercetin, rutin, and anthocyanin derivatives were shown to be more abundant in JIZI 2 vs. JIZI 1. In addition, analysis of unigenes involved in flavonoid, especially anthocyanin biosynthesis pathway, was performed to mine the key genes in flavonoid metabolism of leaves of *Perilla frutescens.* In total, 77 unigenes that encoded 15 enzymes in the flavonoid biosynthesis pathways were studied (Table 1). Analysis of genes involved in flavonoid metabolism showed that 29 key unigenes had different expression levels, including 17 upregulated and 12 downregulated unigenes in JIZI 2 vs. JIZI 1. The core genes in the flavonoid pathway were analyzed in detail, and the results showed the early genes (*PAL*, *CHS*, etc.) or late genes (*DFR*, *ANS*, etc.) had higher expression levels in JIZI 2 vs. JIZI 1. Among the DEGs, 1 *PAL*, 1 *C4H*, 2 *4CL*, 4 *CHS*, 1 *F3H*, 2 *F3′H*, 1 *DFR*, 1 *ANS*, 1 *UAGT*, 1 *UFGT*, and 2 *UGT75C1* genes were upregulated by 1.54 to 5.17-fold (Log_2_FC), whereas 6 *4CL*, 1 *CHI*, 3 *UAGT*, and 2 *FLS* genes were downregulated by -1.14- to -6.24-fold (Fig. 4 and Table S3). These DEGs affected the biosynthesis of flavonoids in the leaves of *Perilla frutescens*.

**Table 1:** Candidate genes related to flavonoid biosynthesis in the leaves of *Perilla frutescens.*

Gene	Enzyme	No. All^a^	No. Up^b^	No. Down^c^
PAL	Phenylalanine ammonia-lyase	3	1	0
C4H	Cinnamic acid 4-hydroxylase	3	1	0
4CL	4-coumarate CoA ligase	15	2	6
CHS	Chalcone synthase	10	4	0
CHI	Chalcone isomerase	7	0	1
F3H	Flavanone 3-hydroxylase	5	1	0
F3*′*H	Flavonoid 3*′*-hydroxylase	2	2	0
F3*′*5*′*H	Flavonoid 3*′*5*′*-hydroxylase	1	0	0
DFR	Dihydroflavonol 4-reductase	4	1	0
ANS/LDOX	Anthocyanidin synthase/leucocyanidin dioxygenase	3	1	0
UAGT	Anthocyanidin 3-O-glucosyltransferase	10	1	3
UFGT	Flavonoid 3-O-glucosyltransferase	5	1	0
FLS	Flavonol synthase	3	0	2
FNS II	Flavone synthase II	2	0	0
UGT75C1	Anthocyanidin 3-O-glucoside 5-O-glucosyltransferase	4	2	0

No. All^a^, the total number of genes.

No. Up^b^, the number of upregulated genes.

No. Down^c^, the number of downregulated genes.

**Figure 5:**
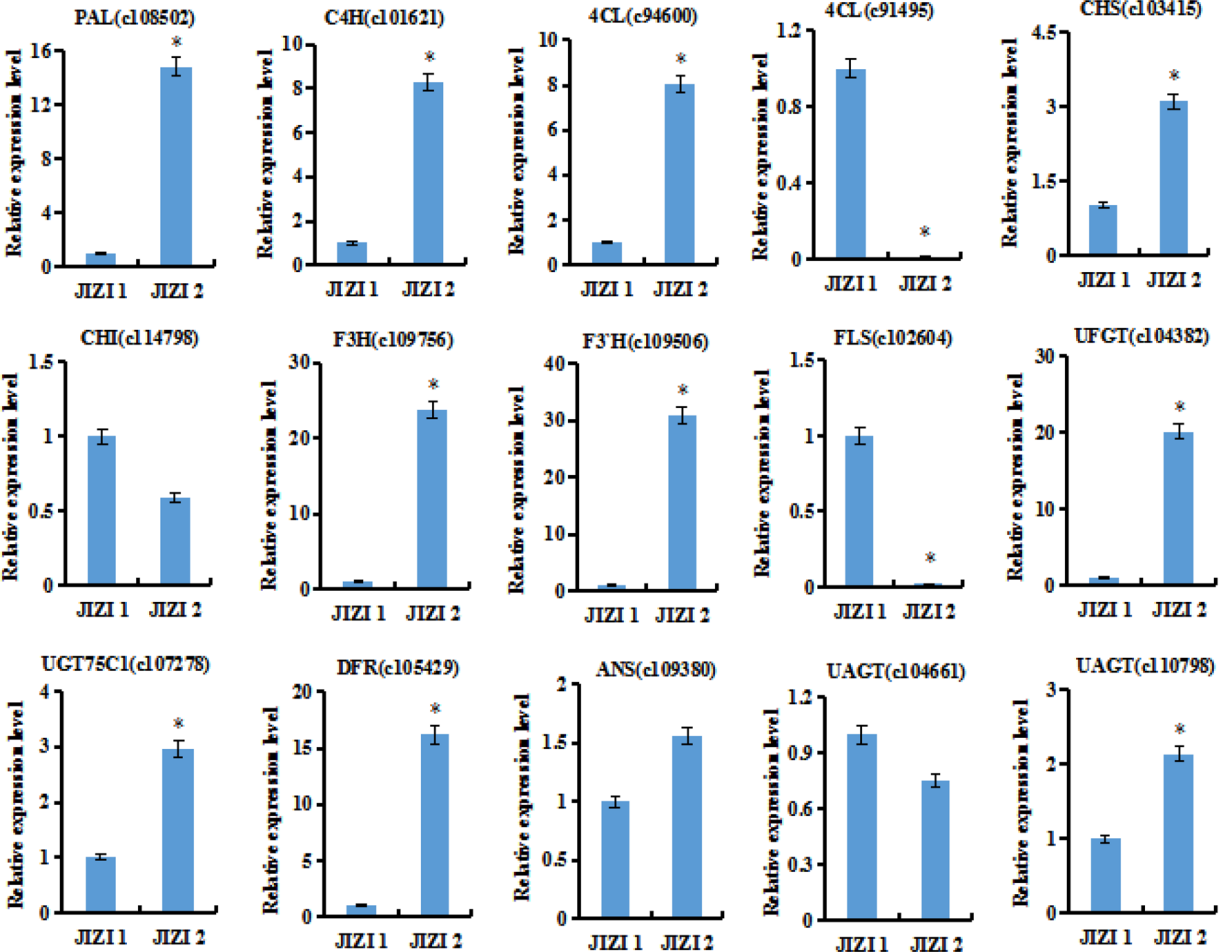
Relative expression levels of 15 genes in flavonoid biosynthetic pathway by qRT-PCR analysis. Data are presented as means ± standard deviations (n = 3).

To verify the credibility of transcriptome information, we further selected 15 DEGs to validate the sequencing results. The qRT-PCR results showed that 11 genes showed higher expression levels, 4 genes were lower expression in JIZI 2 than in JIZI 1, our qRT-PCR results were consistent with those obtained with the RNA-Seq method (Fig. 5).

### Transcription factors of flavonoid biosynthesis in leaves of *Perilla frutescens*

**Figure 6:**
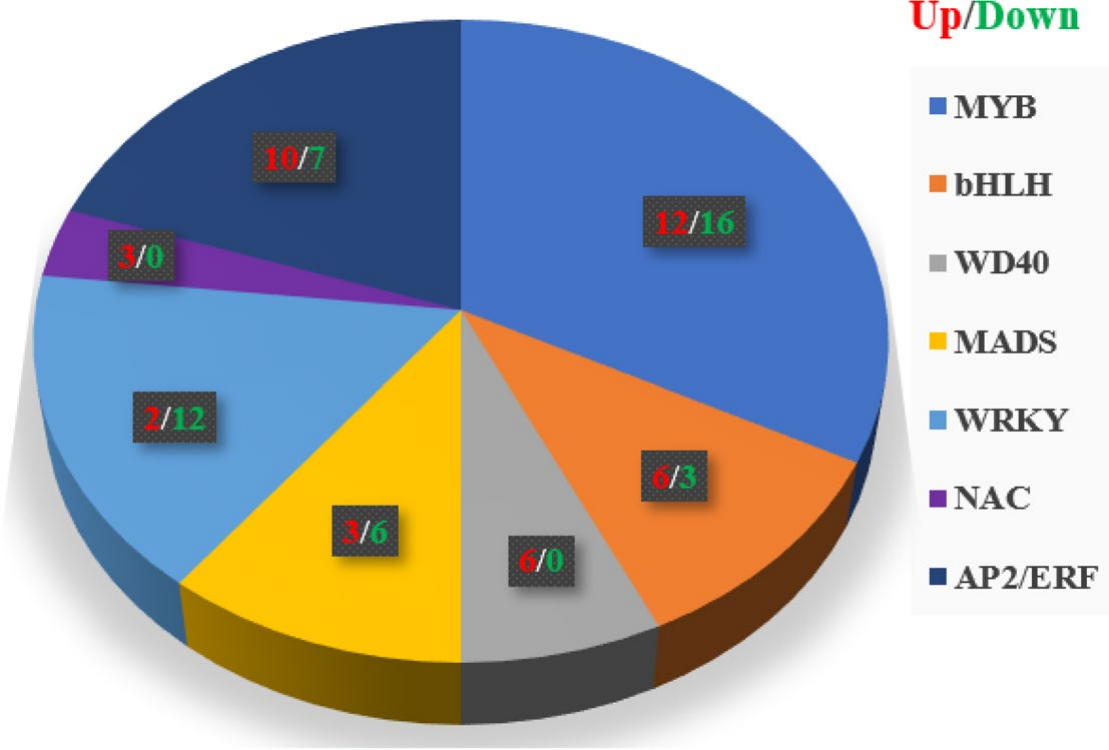
Transcription factors of flavonoids biosynthesis in the leaves *Perilla frutescens*.

Transcription factors participate in flavonoid biosynthesis processes by regulating the gene expression in plants. In our data, we predicted transcription factors using iTAK software (https://itak.feilab.net/). Eighty-six transcription factors with different expression levels were related to flavonoid biosynthesis (Fig. 6 and Table S4). Among these transcription factors, the most abundant were MYBs (28) and AP2/ERFs (17), followed by WRKYs (14), bHLH (9), MADs (9), WD40s (6), and NACs (3). Interestingly, WD40s and NACs were all upregulated. These transcription factors might contribute to flavonoid metabolites in the leaves of *Perilla frutescens*.

## Discussion

UPLC/ESI-Q TRAP-MS/MS is popular in the field of identification and analysis of plant metabolites, which has the advantages of high sensitivity and throughput, fast separation, and wide coverage. So far, this technology is widely applied to analyze the metabolites in tomato, strawberry and asparaguses^16–18^. In recent years, metabolomics integrated with transcriptomics is widely used to investigate the biosynthesis of metabolites to reveal the biosynthesis pathways of metabolites in plants^19^. *Perilla frutescens* (L.) has been used for centuries as a traditional medicinal herb in China. Increasing attention has been given to the anti-allergic, antioxidant, anti-inflammatory, and anti-tumor activities of *perilla* plants^20–24^. It was reported that the flavonoids in *Perilla frutescens* leaves have hypolipidemic and antioxidant effects^25^. In particular, luteolin and apigenin can improve several hyper-monoaminergic neuropsychological disorders as monoamine transporter activators^26^. In our study, the total flavonoid content of JIZI 2 was significantly higher than that of JIZI 1 (Fig. 1). In order to elucidate flavonoid biosynthesis in the leaves of *Perilla frutescens*, metabolomic and transcriptomic data were collected for JIZI 2 and JIZI 1 leaves. A total of 223 flavonoids were identified in the leaves of *Perilla frutescens* by UPLC/ESI-Q TRAP-MS/MS. Of these flavonoids, 57 were significantly different in JIZI 2 vs. JIZI 1, including 46 upregulated flavonoids and 11 downregulated flavonoids (Fig. 2).

Chrysoeriol is a flavonoid metabolite with anti-inflammatory, anti-tumor, and cardioprotective agent effects. Choi et al. reported that chrysoeriol isolated from the leaves of *Digitalis purpurea* could inhibit the induction of nitric oxide synthase by blocking AP-1 activation^27^. Yang et al. reported that chrysoeriol had anti-tumor activity against human multiple myeloma cells^28^. Chrysoeriol could potentially serve as a novel cardioprotective agent against doxorubicin-induced cardiotoxicity^29^. In our study, we found the contents of chrysoeriol and its glycosides were highest in the leaves of *Perilla frutescens*, which were almost 50% of total flavonoid contents. In addition, the apigenin and its derivatives were also abundant in the leaves of *Perilla frutescens*, which are recognized as bioactive flavonoids possessing anti-oxidant, anti-inflammatory, and anti-cancer activities^30^. So far, few studies have qualitatively and quantitatively studied flavonoids in the leaves of *Perilla frutescens* or their biosynthesis pathway. From our data, the skeletons of most flavonoids in *Perilla frutescens* leaves are chrysoeriol, apigenin, hesperetin, quercetin, kaempferol, etc. The most abundant flavonoids in *Perilla frutescens* leaves are O-glycosides or C- and O- diglycosides, with only a few C-glycosides (Table S1).

Combinatorial analysis of transcriptomic and metabolomic are useful for understanding of molecular regulation of secondary metabolites. Fukushima et al. reported that high expression levels of genes encoding F3*′*H, DFR, and ANS leaded to accumulate more anthocyanins in red *perilla* than in green *perilla*^31^. In this study, transcriptome analysis of the leaves of *Perilla frutescens* identified unigenes involved in the flavonoid biosynthesis process and showed differentially expressed genes. In the flavonoid biosynthesis pathway, the expression levels of genes encoding PAL, C4H, CHS, F3H, F3*′*H, DFR, ANS, UFGT, and UGT75C1 were higher in JIZI 2 than in JIZI 1 (Fig. 4) which resulted in a higher flavonoid content in JIZI 2. CHS is a key enzyme of the flavonoid pathway that plays an important role in the phenylpropanoid pathway and in flavonoid biosynthesis. In this study, we detected 4 differentially expressed CHS genes that were all upregulated (Table 1), and metabolic analysis showed that the abundance of naringenin chalcone, which is primarily catalyzed by CHS, was also higher in JIZI 2 than in JIZI 1 (Table S1). Zhang et al. reported that the silenced CHS gene enhanced the phenolic acid content and decreased the accumulation of flavonoids in *Salvia miltiorrhiz*a^32^. Matoušek et al. reported that naringenin chalcone is a precursor of prenylflavonoids and plays an important role in the biosynthesis pathways of flavonoid^33^. Based on the metabolomic and transcriptomic data, we speculated that the high expression of the *CHS* gene and the high abundance of naringenin chalcone enhanced the accumulation of flavonoids in JIZI 2*.*

The biosynthesis of flavonoids is mostly regulated by transcription factors at the transcription level. The MYB-bHLH-WD40 complex, Zinc finger, MADS-box, NACs, and WRKY proteins have been identified to play a role in flavonoid biosynthesis in plants^34–36^. The R2R3-MYB transcription factors regulate the accumulation of flavonol in different parts of *Arabidopsis* seedlings^37^. MYB12 controls flavonol biosynthesis by controlling the expression of CHS, CHI, F3H and FLS^38^. In addition, transcription factor WRKY23 controls flavonol biosynthesis during *Arabidopsis* root development^39^. The expression levels of bHLH transcription factors were significant upregulation in red *perilla* than in green *perilla*^31^. The bHLH factor Myc-F3G which regulated the expression of anthocyanin genes was detected specifically in red *perilla* but not in green *perilla*^40^. In our study, we analyzed the transcriptome data and found that 86 important transcription factors including MYB, AP2/ERFs, WRKY, bHLH, MADS, WD40, and NACs showed significantly different expression levels (Table S4). These differentially expressed transcription factors might be candidate regulators of flavonoid biosynthesis in the leaves of *Perilla frutescens*.

In summary, we found the total flavonoid content was different in the *Perilla frutescens* varieties “JIZI 1” and “JIZI 2”. We used metabolomics and transcriptomics to data reveal the flavonoid biosynthesis metabolic pathway. A total of 9277 differentially expressed genes and 223 flavonoid metabolites were identified in two varieties of *Perilla frutescens*. Chrysoeriol, apigenin, malvidin, cyanidin, kaempferol, and their derivatives were abundant in *Perilla frutescens* leaves. Integrated analysis of transcriptomic and metabolomic data showed that 77 unigenes encoding 15 enzymes that are involved in flavonoid biosynthesis in the leaves of *Perilla frutescens*, among the high expression of the *CHS* gene enhances the accumulation of flavonoids in the leaves of *Perilla frutescens*. Our results provide valuable information on the flavonoid metabolites and the candidate genes involved in the flavonoid biosynthesis pathways in *Perilla frutescens*.

## Materials and methods

### Plant materials

The *Perilla frutescens* varieties “JIZI 1” and “JIZI 2” were grown at the *perilla frutescens* germplasm resource center at the Institute of Cash Crops, Hebei Academy of Agricultural and Forestry Sciences, China. They have different agronomic characters, such as leaf color, thousand kernel weight, and volatile oils. Fresh ripe leaves were collected from healthy plants in September 2019. All materials were frozen by liquid nitrogen and stored at -80 ℃ for RNA and metabolite extraction. All experiments were performed in three biological replicates in this study.

### Measurement of total flavonoid content

Approximately 2.5 g powder of *perilla frutescens* leaves was used to measure the total flavonoid content by the Aluminum nitrate colorimetric method. In brief, 0.5 mL crude extract of leaves was mixed with 5.5 mL of 50% ethanol and l mL of 5% NaNO_2_ solution. Then, 1 mL of 10% Al(N0_3_)_3_ solution was added after 6 min of incubation, and the mixture was incubated for another 6 min. Subsequently, 10 mL of 4% NaOH solution and 7 mL of 50% ethanol were added, and the final volume of the mixture solution was 25 mL. The mixture solution stood for 15 min, and then the absorbance was measured at a wavelength of 506 nm by an ultraviolet spectrophotometer (V-5100B, METASH, Shanghai, China). Rutin was used as a standard solution to prepare a calibration curve, and the results were expressed as rutin equivalent on a dry weight basis^41^.

### Measurement of relative anthocyanin content

*Perilla frutescens* leaves (0.1 g) were ground with 1 ml of methanol (0.1% HCl) and were washed twice into 10 mL centrifuge tubes. The final volume of the samples was 5 mL (including methanol (0.1% HCl)). The tissue homogenates were oscillated for 30 s and centrifuged at 4 °C and 12,000 g for 10 min, and the absorbance of the supernatants was measured at a wavelength of 530 nm using an ultraviolet spectrophotometer (V-5100B, METASH). The relative anthocyanin content was calculated using the following formula: Q = V × A530/M (units/g fresh weight), where V represents the volume of the solution, and M represents the weight of the sample. Methanol (0.1% HCl) was used as a blank control^42^.

### Metabolite extraction

Freeze-dried leaves were crushed using a mixer mill (MM 400, VERDER RETSCH, Shanghai, China) with a zirconia bead for 1.5 min at a frequency of 30 Hz. Then, 100 mg powder was weighed and extracted overnight at 4 °C with 1.0 mL 70% methanol aqueous solution (V/V = 70%). Following centrifugation at 10,000 g for 10 min, the extracts were absorbed by a CNWBOND Carbon-GCB SPE cartridge (250 mg, 3 mL; ANPEL, Shanghai, China, www.anpel.com.cn/cnw) and filtered through a 0.22-μm microfiltration membrane (SCAA-104; ANPEL, Shanghai, China, https://www.anpel.com.cn/) before UPLC-MS/MS analysis.

### Ultra performance liquid chromatography (UPLC) conditions

A UPLC-ESI–MS/MS system (UPLC, Shim-pack UFLC SHIMADZU CBM30A system, Shanghai, Chian, www.shimadzu.com.cn/) was used to analyze the sample extracts. The UPLC analysis was performed under the method of Wang et al.^43^. UPLC: column, Waters (Shanghai, China) ACQUITY UPLC HSS T3 C18 (1.8 µm, 2.1 mm × 100 mm); solvent system, water (0.04% acetic acid): acetonitrile (0.04% acetic acid); gradient program, 95:5 V/V at 0 min, 5:95 V/V at 11.0 min, 5:95 V/V at 12.0 min, 95:5 V/V at 12.1 min, 95:5 V/V at 15.0 min; flow rate, 0.40 mL/min; temperature, 40 °C; injection volume: 2 μL. The effluent was alternatively connected to an ESI-triple quadrupole-linear ion trap (Q TRAP)-MS.

### ESI-Q TRAP-MS/MS

Linear ion hydrazine-flight time (LIT) and triple quadrupole (QQQ) scans were conducted on a triple Q TRAP, API 6500 Q TRAP LC/MS/MS system (Applied Biosystems, Shanghai, China) equipped with an ESI turbo ion-spray interface, operating in positive ion mode and negative ion mode. The system was controlled by Analyst 1.6 software (AB SCIEX, Shanghai, China). The ESI source was set with the following parameters: ion source, turbo spray; source temperature 500 °C; ion spray voltage (IS) 5500 V. The ion source gas I (GSI), gas II (GSII), and curtain gas (CUR) were set at 55.0, 60.0, and 25.0 psi, respectively; the collision gas (CAD) was high. Instrument tuning and mass calibration were performed with 10 and 100 μmol/L polypropylene glycol solutions in QQQ and LIT modes, respectively. QQQ scans were acquired as multiple reaction monitoring (MRM) experiments with collision gas (nitrogen) set to 5 psi. Declustering potential (DP) collision energy (CE) measurements for individual MRM transitions were completed with further DP and CE optimization. A specific set of MRM transitions was monitored for each period according to the metabolites eluted within the period^44^.

### Identification and quantitative analysis of metabolites

Qualitative analysis of the metabolite data was performed base on the Metware Database (Metware Biotechnology Co., Ltd. Wuhan, China). A home-made software reference to MetDNA of Zhu lab and Masterview from AB Sciex were used to give the matching score of metabolites with the Metware Database. Most compounds of the database were standards. The metabolites for which no standards were available, peaks in the MS2T library, mainly the peaks that showed similar fragmentation patterns with the identified metabolites, were used to query the MS 2 spectral data taken from the literature or to search the public metabolite databases (such as MassBank, KNAPSAcK, HMDB, MoToDB, and METLIN)^45^. The quantitative analysis of metabolites used multiple reaction monitorin^46^. In the MRM mode, the precursor ions were fragmented after the induced ionization of the collision chamber to form many fragment ions, then selected a characteristic fragment ion by triple quadrupole filtering to eliminate the interference of non-target ions. After obtaining the metabolite mass spectrometry data of different samples, the peak area of all substance mass peaks was integrated, and the peaks of the same metabolite in different samples were integrated and corrected.

An unsupervised PCA (principal component analysis), HCA (hierarchical cluster analysis), and OPLS-DA (partial least squares-discriminant analysis) were performed within R^47^. The unsupervised PCA was performed by statistics function prcomp within R (www.r-project.org), and the data was unit variance scaled before unsupervised PCA. The HCA results of samples and metabolites were presented as heatmaps with dendrograms, while pearson correlation coefficients (PCC) between samples were caculated by the cor function in R and presented as only heatmaps. Both HCA and PCC were carried out by R package pheatmap (version 1.0.12). For HCA, normalized signal intensities of metabolites (unit variance scaling) were visualized as a color spectrum. Significantly different metabolites between groups were determined by VIP ≥ 1 and an absolute Log_2_FC (fold change) ≥ 1. VIP (variable importance in project) values were extracted from OPLS-DA result, which also contained score plots and permutation plots, were generated using R package MetaboAnalystR. The data was log transform (log_2_) and meant centering before OPLS-DA. In order to avoid overfitting, a permutation test (200 permutations) was performed.

### RNA extraction and Illumina sequencing

Total RNA was extracted from frozen leaves using the RNAprep Pure Plant Kit (TIANGEN Biotech, Beijing, China). RNA degradation and contamination were monitored on 1.2% agarose gels. The purified RNA concentrations were quantified using a spectrophotometer (ThermoFisher Scientific, Shanghai, China). The quality of the total RNA was exaNanoDrop 2000 mined using an Agilent 2100 Bioanalyzer (Agilent Technologies, Santa Clara, CA, USA). Poly (A) mRNA was enriched from the total RNA using Oligo (dT) magnetic beads. Poly (A) mRNA was subsequently fragmented by an RNA fragmentation kit (Ambion, Austin, TX, USA). The fragmented RNA was transcribed into first-strand cDNA using reverse transcriptase and random hexamer primers. Second-strand cDNA was synthesized using DNA polymerase I and RNase H (Invitrogen, Carlsbad, CA, USA). After end repair and the addition of a poly (A) tail, suitable length fragments were isolated and connected to the sequencing adaptors. The fragments were sequenced on an Illumina HiSeq 2500 platform^48^.

### RNA sequencing (RNA-seq) data analysis and annotation

To acquire high-quality reads, the raw reads in fastq format were processed through in-house Perl scripts. Clean reads were obtained from raw data by removing adaptor sequences, low-quality reads, and reads containing ploy-N. All downstream analyses were based on clean data with high quality. Transcriptome assembly was accomplished using Trinity software (version2.5.1)^49^. Gene function was annotated using the following: the Kyoto Encyclopedia of Gene and Genome (KEGG) pathway database (https://www.genome.jp/kegg), the NCBI non-redundant (Nr) database (version 2018.4), the Swiss-Prot protein database (https://www.uniprot.org), the euKaryotic Clusters of Orthologous Groups (KOG) database (version 1.0), the Gene Ontology (GO) database (https://www.geneontology.org), and the Pfam database (version 33.0).

The levels of gene expression were estimated by RSEM (version 1.2.26)^50^. Analysis of the differentially expressed genes of the two groups was performed with the DESeq R package (1.10.1). DESeq provides statistical routines for determining differentially expressed genes using a model based on the negative binomial distribution. The results of all statistical tests were corrected by multiple testing using the Benjamini and Hochberg false discovery rate. Genes were determined to be significantly differentially expressed at an adjusted *P* value < 0.05 according to DESeq. GO enrichment analysis of the differentially expressed genes was implemented by the topGO R package based on the Kolmogorov–Smirnov test. Pathway analysis elucidated significant pathways of differentially expressed genes according to the KEGG database (https://www.genome.jp/kegg)^51–53^. We tested the statistical enrichment of differentially expressed genes in KEGG pathways using KOBAS software^54^. The whole set of raw data can be found in the national center for biotechnology information (NCBI) SRA database (accession number SRP270356).

### QRT-PCR expression analysis of genes involved in anthocyanin biosynthesis

Total RNA of *Perilla frutescens* leaves was reverse-transcribed according to the Quantscript Reverse Transcriptase Kit (TIANGEN Biotech, Beijing, China). cDNA was used as a template to measure gene expression. The specific primers involved in the anthocyanin biosynthesis genes and the *Perilla frutescens* actin gene (internal control) are listed in Table S5. Quantitative real-time polymerase chain reaction (qRT-PCR) was conducted by a real-time PCR ABI Prism 7500 system (software for 7500 and 7500 Fast Real-Time PCR Systems, V2.0.1, Foster City, CA, USA) using SYBR Premix Ex Taq II (TaKaRa Code No. RR820A, https://www.takara biomed.com.cn). The comparative CT method (2^−ΔΔCT^ method) was used to quantify gene expression^55^.

### Statistical analysis

Statistical analysis was performed using Excel 2010 software (Microsoft Office, USA). Data are presented as means ± standard deviations (SD). The levels of statistical significance were analyzed by the least significant difference (*p* < 0.05).

## Supplementary information

Below is the link to the electronic supplementary material.

Supplementary information.

Supplementary Legends.

Supplementary Tale S1.

Supplementary Tale S2.

Supplementary Tale S3.

Supplementary Tale S4.

Supplementary Tale S5.
