# Supplementary material for: Integrated transcriptomic and metabolomic data reveal the flavonoid biosynthesis metabolic pathway in Perilla frutescens (L.) leaves
Source: Sci Rep. 2020 Oct 1;10:16207. doi: 10.1038/s41598-020-73274-y (PMC7530993; doi:10.1038/s41598-020-73274-y)
Supplement: Supplementary file 2 — Supplementary Legends. [file 41598_2020_73274_MOESM2_ESM.docx]

**Supplementary Material**

**Integrated** **Transcriptomic and** **Metabolomic Data Reveal** **the** **Flavonoid** **Biosynthesis Metabolic Pathway in** ***Perilla frutescens* (L.) Leaves**

Tao Jiang ^1†^, Kunyuan Guo ^2†^, Lingdi Liu ^1^, Wei Tian^1^, Xiaoliang Xie^1^, Saiqun Wen^1^, Chunxiu Wen^1^*

1 Institute of Cash Crops, Hebei Academy of Agricultural and Forestry Sciences, Shijiazhuang, 050051, Hebei, China

2 Institute of Chinese Herbal Medicines, Hubei Academy of Agricultural Sciences, Enshi, 445000, Hubei, China

*Corresponding authors E-mail: [chunxiuwen@163.com](mailto:chunxiuwen@163.com)

†These authors contributed equally to this work.

Supplementary Table S1 Flavonoid metabolites in the leaves of *Perilla frutescens*

Supplementary Table S2 *Perilla frutescens* All_Database_annotation

Supplementary Table S3 Core genes in the flavonoid pathway

Supplementary Table S4 Transcription factors of flavonoid biosynthesis in leaves of *Perilla frutescens*

Supplementary Table S5 Sequences of specific primers for qRT-PCR

Supplementary *Perilla frutescens* unigenes sequence
